# Combined Spectroscopic Methods of Determination of Density of Electronic States: Comparative Analysis of Diffuse Reflectance Spectroelectrochemistry and Reversed Double-Beam Photoacoustic Spectroscopy

**DOI:** 10.1021/acs.jpclett.1c00262

**Published:** 2021-03-18

**Authors:** Marcin Kobielusz, Akio Nitta, Wojciech Macyk, Bunsho Ohtani

**Affiliations:** † Faculty of Chemistry, Jagiellonian University, ul. Gronostajowa 2, 30-387 Kraków, Poland; ‡ Institute for Catalysis, Hokkaido University, Sapporo 001-0021, Japan; § Graduate School of Environmental Science, Hokkaido University, Sapporo 060-0810, Japan

## Abstract

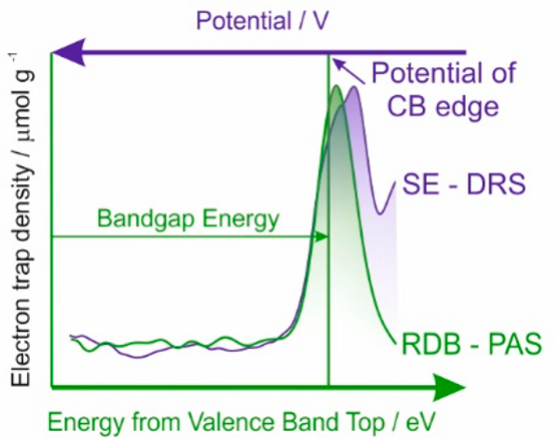

The diffuse reflectance spectroelectrochemistry (SE-DRS) and reversed double-beam photoacoustic spectroscopy (RDB-PAS) provide unique, complementary information on the density of electronic states (DOS) in the vicinity of the conduction band bottom. The measurements are performed under quite different conditions, representing the solid/liquid and solid/gas interfaces in SE-DRS and RDB-PAS, respectively. DOS profiles obtained from both types of measurements can be considered as unique “fingerprints” of the tested materials. The analysis of DOS profiles recorded for 16 different TiO_2_ samples confirms that both methods similarly describe the shapes of DOS profiles around the conduction band edges. The states characterized by energy higher than VBT (valence-band top) + *E*_g_ can be considered as electronic states within the conduction band. Recognition of the potential of the conduction band bottom allows one to classify the electronic states as deep or shallow electron traps or conduction band states, which play different roles in photocatalysis. The comparative analysis shows that both methods provide very useful information which can be used in understanding and predicting the photo(electro)catalytic reactivity of semiconductors.

The activity of heterogeneous photocatalysts strongly depends on their physicochemical properties, such as band gap energy, potential of the conduction and valence band edges, phase composition,^1−4^ surface area,^5,6^ particle size,^7−9^ and facet exposition.^10,11^ Therefore, usually the characteristics of materials is focused on examining these parameters. Nevertheless, the influence of other factors on photocatalytic activity, especially the presence of various defects in the crystal structure, cannot be neglected. For instance, Wang et al. showed that the separation efficiency of photogenerated electrons and holes could be significantly improved by adjusting the bulk/surface defects ratio.^12^ Particularly surface defects seem to play a pivotal role since the surface is a scene of the most important photocatalytic processes. Defects (e.g., oxygen vacations) cannot only facilitate binding of substrates and act as active sites but also affect the electronic structure of semiconductors.^13^ They can act as traps of photogenerated holes and electrons. In this way, they can influence the availability and recombination efficiency of charges as well as determine the effective semiconductor redox potential.^14,15^ Therefore, the ability to determine the energy and density of electronic states (DOS) relative to the conduction band and a reference potential (e.g., SHE) is of paramount importance.

Reversed double-beam-photoacoustic spectroscopy (RDB-PAS) has been developed as a unique spectroscopic-analysis method enabling identification and detailed characterization of semiconducting materials, not limited to photocatalysts, by getting a fingerprint of metal oxides and the other semiconducting materials.^16,17^ The principle is that vacant electronic states in the bandgap, electron traps (ETs), are filled by direct energy (wavelength)-selective photoexcitation of electrons in the valence band (VB) from the deeper (longer wavelength) side to the shallower side by wavelength-scanned continuous monochromatic light irradiation, and an increase in photoabsorption by accumulation of electrons in ETs is measured by photoacoustic spectroscopy using modulated (continual) LED-light. Then the obtained spectrum is differentiated from the longer-wavelength (lower energy) side to the shorter-wavelength (higher energy) side to obtain the energy-resolved distribution of ETs (ERDT), i.e., the DOS profile for vacant electronic states in the bandgap. The obtained density recorded in arbitrary units is converted in the unit of μmol g^–1^ by comparing the reported results of chemical titration of ETs.^18^ The energy of ETs, determined from the wavelength of the excitation light, is presented as energy measured from the VB top (VBT) of each sample. Since the density of ETs in samples is, in general, very low or almost negligible, the actual photoexcitation of VB electrons to ET must occur from the high density-of-states (DOS) part of VB, not VB top, where the DOS is almost zero and the energy of ERDT is overestimated.^19^ For titania samples, this overestimation was suggested to be 0.1–0.2 eV.^16,17^ This is the reason why a part of ETs are found in a conduction band (CB) in ERDT, since the plotted CB bottom position is estimated by measuring the absorption edge wavelength, corresponding to the bandgap energy and shown in the same energy scale, energy with reference to VB top (same as ERDT). At present we have no possible way to calibrate the difference in energy between VB top and a high-DOS part. In this sense, if this energy difference varies depending on the bulk crystalline (amorphous) structure, discussion on the difference in ERDT of different samples is not straightforward. The influence of this problem will be discussed later.

Another feature of ETs detected by RDB-PAS is that they are mainly located on the surface since a plot of total ET density versus specific surface area of commercial titania samples shows the trend of almost proportional relation. The rough estimation of density of ETs in commercial titania samples was ∼1 ET nm^–2^,^17^ suggesting that ETs detected by RDB-PAS might not be point defects but buried in a reconstructed surface structure or on ridges (steps) of crystalline facets.

Recently, we have developed a spectroelectrochemical method which combines UV–vis diffuse reflectance spectroscopy and electrochemistry (SE-DRS).^20,21^ In the proposed method, the studied powder material is casted on the surface of a platinum plate (working electrode). A progressive, negative bias of the electrode leads to the electrochemical reduction of the material. This should be understood as a gradual filling of unoccupied electronic states. For instance, electrochemical reduction of TiO_2_ leads to the formation of titanium(III) centers, characterized by a broad absorption band with a maximum localized at 780 nm.^22^ Both in the SE-DRS and RDB-PAS methods, reduction of Ti(IV) plays a crucial role. Simultaneous observation of the cathodic current and Kubelka–Munk function changes (ΔKM) allows an unambiguous determination of the potential of the electron state. Furthermore, quantification of the Kubelka–Munk function alterations between consecutive reduction sequences gives information on the relative concentration of available states.

Electrochemical reduction of electronic states allows the measurement of the effective redox potential of electronic states. The possibility of DOS determination as the function of redox potential makes this method independent of the precise determination of the band edges energy.^23−26^ The electrochemical approach can be directly applied to real, also nanocrystalline, samples, there is no need to neglect the existence of electronic states other than bands or to assume that the studied material consists of almost ideal crystals.^27^ This advantage of the SE-DRS method allows one to compare, *inter alia*, the redox properties of different semiconductors, their polymorphs, or (surface) modifications.^2,28−32^

Electrochemical measurements require the use of electrolytes, which impose an applicable potential range and can influence the surface properties of the studied material. Redox properties of semiconductors depend to some extent on pH, solvent polarity, presence of various cations and anions, *etc*. Meyer et al.^33^ have shown that the flat band potential may be shifted in the presence of some cations, which may penetrate the crystal lattice. The use of the photochemical reduction method (here RDB-PAS) solves some of these problems. First of all, the use of electrolytes is not required, and the range of available energies (potentials) depends in this case only on the energy of photons. However, a hole scavenger has to be involved. The ability to reduce states depends on the reduction rate (activation energy) and the oxidation rate of the scavenger. The use of primary alcohol as a scavenger significantly facilitates the reduction of electronic states close to the conduction band due to the photocurrent doubling effect.

SE-DRS and RDB-PAS methods together provide more information than any of them applied alone. Both methods, due to the distinct differences described above, may give slightly different results. Therefore, it is important to understand these differences and their origins. Here we present a comparison of data taken with SE-DRS and RDB-PAS methods for a series of commercially available titanium(IV) oxide materials.

Comparative measurements were carried out for samples of titanium dioxide differing, among others, in the phase composition, the specific surface area, and bandgap energy. Selected physical properties are summarized in Table 1.

**Table 1:** Physical and Structural Properties of Studied TiO_2_

	phase composition/%^a^			
sample	anatase	rutile	specific surface area/m^2^ g^–1^	bandgap energy/eV
TIO-3	0	90	47	2.98
Tronox TR	0	86.5	5.5	2.96
MT-150A	0	82	114	3.03
TiO-6	0	78	102	3.03
TIO-13	93	0	70	3.19
TIO-1	91	0	79	3.20
TiO-2	91	0	17	3.14
ST-01	80	0	344	3.17
Tronox AK-1	74.6	0	90	3.17
CR-EL	1	94	8	2.97
ST-G2	3	95	4	2.95
TiO-5	9	85	6	2.96
ST-F1	78	20	22	3.02
TiO-11	82	9	100	3.14
P25	82	9	58	3.06
ST-F5	84	3	84	3.16

a The phase composition was determined by Rietveld analysis of XRD patterns using nickel oxide as an internal standard, following the previous paper with the same instrumental setups.^34^

The density of electronic states obtained with RDB-PAS and SE-DRS are determined relative to different references, which are either the energy of the top of the valence band or the electrochemical potential scale, respectively. Therefore, comparison of these data is not straightforward. Here we propose to assume that the main (left) slopes of the graph, i.e., the increase of the density of the electronic states associated with the lower edge of the conduction band, should overlap. In this way, the results collected with RDB-PAS and SE-DRS were compared in Figure 1.

**Figure 1:**
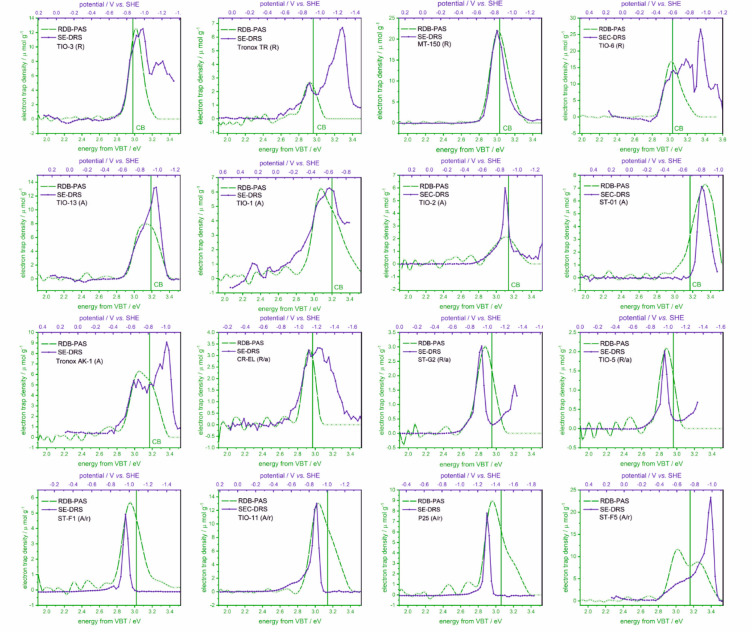
Distribution of electronic states determined for 16 TiO_2_ samples with RDB-PAS (green) and SE-DRS (blue) methods. The green lines show the determined bandgap energy versus VBT.

DOS profiles collected in Figure 1 show some clear similarities. In the case of the materials composed mainly of anatase, the main slopes appear at potentials of −0.4 to −0.8 V vs SHE, while for rutile samples the onset potential is shifted cathodically. After reaching a maximum, the DOS function decreases at low potentials (high energies). For instance, for TiO-3, such DOS reduction is observed above 3.0 eV from VBT (−1.9 V vs SHE). Also, the main differences in the density of states determined by RDB-PAS and SE-DRS appear in this range. A significant lowering of DOS at higher energies can be attributed to the difficulties in the extensive reduction of electronic states, usually localized within the conduction band. This decrease can be observed, e.g., for the MT-150A sample for which SE-DRS measurements were taken for two electrodes covered with different amounts of the material (Figure 2a). For the thicker TiO_2_ film, only one intense maximum was observed. However, when a thinner film was deposited at the platinum plate, two distinct maxima were recorded. The second, higher maximum appeared in the energy range where for the thicker film almost no signal was recorded. A similar effect was observed when the Kubelka–Munk function change was analyzed at different wavelengths (Figure 2b). At longer wavelengths, the differences were negligible; however, at shorter wavelengths the absorption coefficient of Ti(III) decreases and therefore the shape of the recorded curves at high energies (low potentials) changed significantly. This effect is even more pronounced when the curves are normalized (the same intensity of the maximum; Figure 2c). The second, high energy maximum becomes pronounced. This analysis points at a limited sensitivity of the SE-DRS approach, which depends on the film thickness and the wavelength at which the analysis is performed.

**Figure 2:**
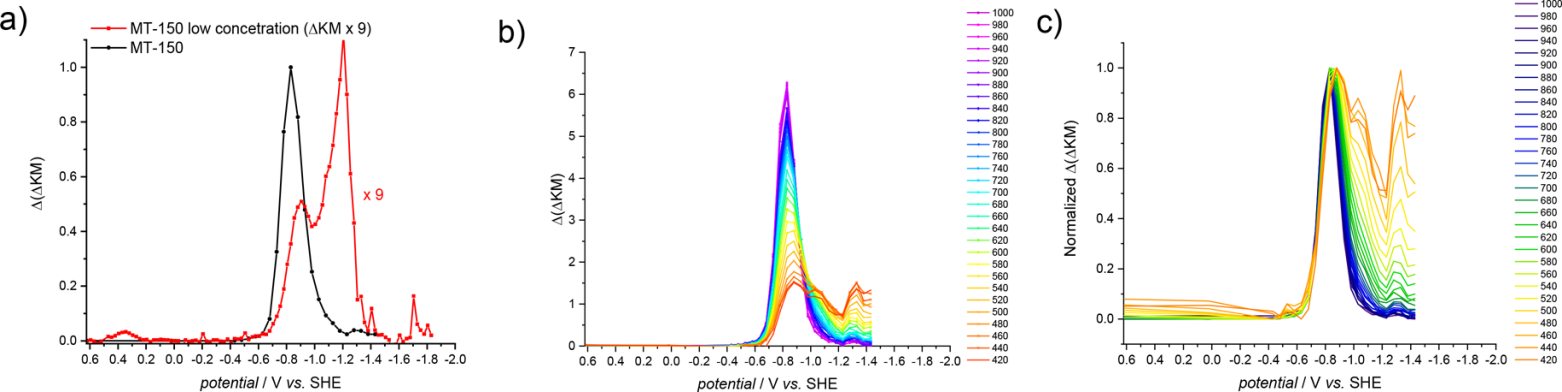
SE-DRS measurements collected for the MT-150A sample casted at the Pt electrode: the influence of the sample thickness (a) and the detection wavelength before (b) and after (c) normalization.

The difference between SE-DRS and DB-PAS results originates also from various measurement conditions. In the former case, an electrolyte is applied, while in the latter one the sample is dry. In the presence of aqueous and anhydrous electrolytes the distribution of electronic states may vary due to different influences of electrolytes on the surface of the semiconductor (changes in polarity, presence of various ions, adsorption/desorption processes, *etc.*). In particular, in aqueous electrolytes the equilibria between adsorbed/desorbed H^+^ and OH^–^ ions are established, which influence the flat band potential.^27^ In nonaqueous solutions, such as acetonitrile, the flat band potential is shifted toward lower potential values compared to the aqueous electrolytes.^35^ This shift strongly depends on the ions present in the solution. In particular, lithium cations have a similar effect to protons in protic electrolytes. Enright et al. demonstrated that in a solution containing 0.1 M Li^+^, the flat band potential of polycrystalline TiO_2_ is shifted to −0.90 V from −0.82 V vs SCE measured in the aqueous electrolyte (pH = 7).^36^ The influence of these ions on DOS determined by SE-DRS manifests mainly in shifting the DOS function along the potential axis, while the shape of the function remains nearly unchanged.^20^ The presence of water in the electrolyte causes a significant decrease in the recorded signals intensity, which makes it difficult to observe any other effects of this solvent on DOS. However, measurements of SE-DRS in the acetonitrile electrolyte containing small amounts of water (e.g., 1:19) show that the DOS contours determined in aqueous and nonaqueous electrolytes are not completely different (Figure 3).

**Figure 3:**
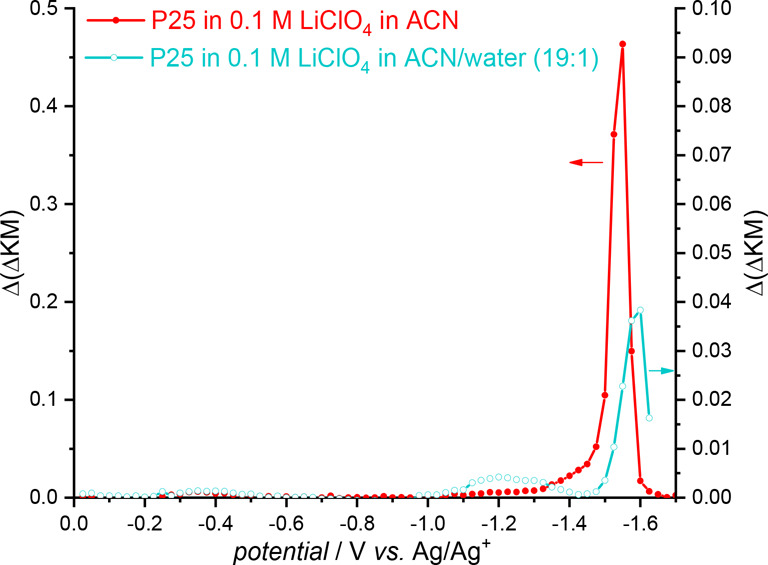
Density of electronic states determined for P25 in electrolyte solution containing 0.1 M LiClO_4_ in acetonitrile (red) and in the mixture of acetonitrile and water (19:1; blue).

Despite the aforementioned reasons for which SE-DRS and RDB-PAS methods may give different DOS profiles, for the energy range from the top of the valence band up to energy equal to *E*_g_, the profiles are quite similar. The states characterized by energy higher than VBT + *E*_g_ can be considered as electronic states within the conduction band. Within this region, bigger differences between SE-DRS and RDB-PAS contours appear; however, these states usually play a negligible role in photocatalytic activity of the materials. Notably, during both measurements, the material is strongly reduced. This increases the concentration of titanium(III) species and does not remain neutral also to the whole material, including its stoichiometry, defects, and crystal structure. Recognition of the potential of the conduction band bottom allows one to classify the electronic states as deep or shallow electron traps or conduction band states.

In photocatalysis, electronic states localized within the bandgap and close to CB and VB edges play a key role. According to the Kasha rule, electrons excited to higher energy states, within CB (or holes generated below the maximum of VB), usually will relax to the states (traps) close to the band edges before taking part in any interfacial electron transfer process. Thus, some discrepancies between the DOS profiles within the conduction band resulting from the application of different approaches (SE-DRS and RDB-PAS) do not exclude the applicability of any of them.

Information from the comparison of contours resulting from SE-DRS and RDB-PAS is summarized in Table 2. As already mentioned, the peak position in RDB-PAS is evaluated in reference to VBT (relative values), but that in SE-DRS is shown in reference to the standard hydrogen-electrode potential (SHE; absolute values). First, the formal difference, Δ*E* (i.e., the VBT potential), was calculated. This value can be considered as the conduction band bottom for pure rutile and anatase materials. Then, three kinds of corrections were applied to Δ*E*. (1) As stated in the early part of this paper, ET energy is shown in reference to VBT, for convenience, but actual excitation of electron in VB to ETs may occur from the high-DOS part of VB, which is lower than VBT. This overestimation of ET energy was assessed to be ∼−0.15 eV and applied to all the data of Δ*E*. (2) For the mixture of anatase and rutile, the value of Δ*E* is calculated from the determined bandgap energy and is a resultant of the energies of the conduction band bottoms for pure rutile and anatase materials. Furthermore, it has been proposed that interfacial charge-transfer excitation (ICTE) from the high-DOS part of rutile VB to anatase ETs occurs and the energy of high-DOS part of rutile is ∼0.20 eV higher than that of anatase resulting in the shift of the anatase peak to lower energies.^19^ This correction is applied to anatase–rutile mixed samples. (3) It has been suggested that samples of high specific surface area (>80 m^2^ g^–1^) may contain an amorphous surface layer with a large band gap (shorter-wavelength absorption edge) to make the ET energy slightly higher (cf. Table 1). Approximated value of 0.1 eV (0.2 eV for ST-01 with an extra high specific surface area) was corrected for those samples. After application of those corrections, corrected formal energy difference, Δ*E*_corr_ (i.e., the corrected VBT potential) was ranged at ∼1.9–2.1 V and the average was 2.02 V. The exceptions are P25, TIO-1, and ST-F5. For P25, the anatase–rutile composite, the energy diagram seems to be shifted to lower potentials compared with the other anatase samples,^37^ and hence the correction (2) might be underestimated. It is worth mentioning that fitting of SE-DRS and RDB-PAS profiles may be imperfect and should take into account the best overlapping of the slopes of profile onsets (Figure 4). The proper description of P25 composite should take into account this effect. The ionization potential of TIO-1 corresponding to its VBT evaluated using photoelectron spectroscopy was exceptionally high, 7.88 eV, compared with those of the other titania samples, equal to 7.3–7.5 eV. Since TIO-1 is known to be contaminated with sulfate anions on its surface, which increases surface acidity, it can be presumed that its band diagram is shifted to lower energy.^37^

**Figure 4:**
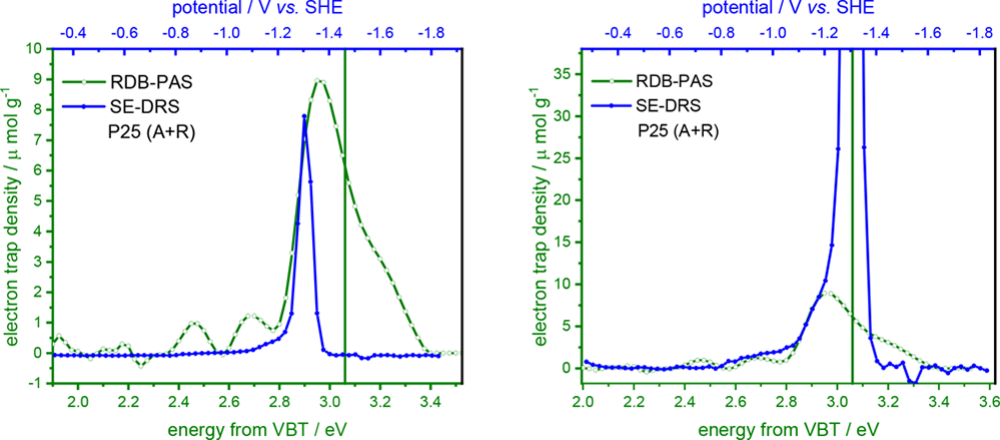
Two ways of comparison of RDB-PAS and SE-DRS profiles presented for P25 taking into account similarities in different parts of the slopes.

**Table 2:** Information from the Comparison of Contours Resulting from SE-DRS and RDB-PAS

titania^a^	crystal^b^	bandgap energy (eV)	SE-DRS (V vs SHE)	Δ*E*^c^ (V vs SHE)	correction (1)^d^	correction (2)^e^	correction (3)^f^	Δ*E*_corr_ (V vs SHE)
TIO-3	R	2.98	–0.88	2.10	–0.15	0		1.95
(T)TR	R	2.96	–0.92	2.04	–0.15	0		1.89
MT-150A	R	3.02	–0.85	2.17	–0.15	0	–0.10	1.92
TIO-6	R	3.02	–0.60	2.42	–0.15	0	–0.10	2.17
TIO-13	A	3.19	–0.91	2.28	–0.15	0		2.13
TIO-1	A	3.20	–0.62	2.58	–0.15	0		2.43
TIO-2	A	3.14	–0.90	2.24	–0.15	0		2.09
ST-01	A	3.17	–0.68	2.49	–0.15	0	–0.20	2.14
(T)AK-1	A	3.17	–0.81	2.36	–0.15	0	–0.10	2.11
CR-EL	R/a	2.97	–1.15	1.82	–0.15	0.20		1.87
ST-G2	R/a	2.95	–1.04	1.91	–0.15	0.20		1.96
TIO-5	R/a	2.96	–1.06	1.90	–0.15	0.20		1.95
ST-F1	A/r	3.02	–1.08	1.94	–0.15	0.20		1.99
TIO-11	A/r	3.14	–1.00	2.14	–0.15	0.20	–0.10	2.09
P25	A/r	3.05	–1.46	1.59	–0.15	0.20		1.64
ST-F5	A/r	3.17	–0.75	2.42	–0.15	0.20	–0.10	2.37
								2.02^h^

a (T), Tronox.

b R, rutile (major); A, anatase (major); r, rutile (minor); and a, anatase (minor).

c Potential of VBT calculated as (bandgap energy) + (SE-DRS).

d Correction for overestimation of CBB position by VBT and high DOS part (= –0.15 eV) in RDB-PAS measurement.

e Correction for underestimation of CBB position by interfacial charge-transfer excitation (ICTE) in anatase-rutile mixture (= 0.20 eV) in RDB-PAS measurement.

f Correction for widened band gap due to surface amorphasization (= ∼–0.10 eV) in RDB-PAS measurement.

g Average of Δ*E*_corr_ values.

The discussed SE-DRS and RDB-PAS methods give information on the distribution of electronic states, which play a pivotal role in photocatalytic activity of the studied semiconductors. The measurements are performed under quite different conditions, representing the solid/liquid and solid/gas interfaces in SE-DRS and RDB-PAS, respectively. DOS profiles obtained from both types of measurements can be considered as unique “fingerprints” of the tested materials. Presented analysis of DOS profiles recorded for 16 different TiO_2_ samples confirm that both methods similarly describe the shapes of DOS profiles around the conduction band edges. Application of both, complementary methods enable the determination of the potential of the conduction band bottom and recognition of the character of electronic states. What is important, both methods support information on redox properties of the semiconductors both in their ground and excited states. This knowledge is very important in understanding and predicting the photocatalytic reactivity.

## Experimental Methods

### RDB-PAS

RDB-PAS analyses were operated on laboratory-made setups to obtain ERDT/CBB (conduction-band bottom) patterns with the following reported procedure,^16,17^ with some modification as follows. A stainless-steel sample holder was loaded with a sample powder and sent in a PAS cell equipped with a MEMS (microelectro-mechanical system; SparkFun MEMS Microphone Breakout, INMP401 (ADMP401)) microphone module, a quartz window, and PVK (polyvinylcarbazol) bulbs. Then, methanol-saturated high-purity (4N) nitrogen gas was made to flow through the cell. Methanol was used as a scavenger of positive holes avoiding disappearance of once-trapped electrons by capturing positive holes. A sample in the cell was irradiated from the upper side with wavelength-scanned monochromatic (pump) light from a grating monochromator with a xenon lamp to excite valence-band electrons directly to electron traps (ETs), and photoabsorption of electrons accumulated in the ETs were monitored by photoacoustic signal with an intensity-modulated (35 or 80 Hz) 625 nm LED (probe) light. By scanning the pump-light wavelength from longer wavelength (650 nm) to shorter wavelength (300 or 350 nm) with a 5 nm step, the ETs in the sample were filled from the deeper (lower energy) side to the shallower (higher energy) side. The thus-obtained RDB-PA spectrum was then differentiated from the lower-energy side to obtain energy-resolved distribution of ETs with density in arbitrary units. Calibration of absolute ET density was made in reference to the previously reported chemical titration study on various titania particles^18^ assuming all the detected ETs were originated from titania. CBB was determined by ordinary single-beam PAS using the same instruments following the procedure reported previously.^17^

### SE-DRS

The SE-DRS measurements were carried out in the three-electrode setup with platinum wire and Ag/Ag^+^ electrode [AgNO_3_ (10 mmol dm^−3^) in 0.1 mol dm^−3^ tetrabutylammonium perchlorate in acetonitrile] as the counter and reference electrodes, respectively. Studied TiO_2_ samples deposited onto platinum foil (∼1 × 3 cm^2^) were used as working electrodes. The electrodes were placed in a cuvette with a quartz window filled with 0.1 mol dm^−3^ LiClO_4_ solution in acetonitrile. The cuvette was placed in front of the integrating sphere (5 cm diameter), facing the working electrode toward the light beam. Oxygen was thoroughly removed from the electrolyte by purging it with argon before (15 min) and during experiments. The electrode potential was controlled by the electrochemical analyzer (Bio-Logic, SP-150). The applied potential was lowered every 10 min by 50 mV. The relative reflectance changes (at 780 nm) were collected by PerkinElmer UV–vis Lambda 12 spectrometer. The relative reflectance changes were converted to the Kubelka–Munk function (ΔKM). Finally, the density of states (DOS) was calculated as a difference in the Kubelka–Munk function between two consecutive potentials. The detailed experimental procedure of SE-DRS has been described and discussed elsewhere.^20^
